# Clinical outcomes of locked plating of distal femoral fractures in a retrospective cohort

**DOI:** 10.1186/1749-799X-8-43

**Published:** 2013-11-27

**Authors:** Martin F Hoffmann, Clifford B Jones, Debra L Sietsema, Paul Tornetta, Scott J Koenig

**Affiliations:** 1 Grand Rapids Medical Education Partners, 1000 Monroe Ave NW, Grand Rapids MI 49503, USA; 2 Universitaetsklinikum Bergmannsheil, Bürkle-de-la-Camp-Platz 1, 44789, Bochum, Germany; 3 Michigan State University/Orthopaedic Associates of Michigan, 230 Michigan St. NE, Grand Rapids MI 49503, USA; 4 Boston Medical Center, 88 East Newton Street, Boston MA 02118, USA

**Keywords:** Femur, Fracture, Supracondylar, Locked plating, Outcome

## Abstract

**Purpose:**

Locked plating (LP) of distal femoral fractures has become very popular. Despite technique suggestions from anecdotal and some early reports, knowledge about risk factors for failure, nonunion (NU), and revision is limited. The purpose of this study was to analyze the complications and clinical outcomes of LP treatment for distal femoral fractures.

**Materials and methods:**

From two trauma centers, 243 consecutive surgically treated distal femoral fractures (AO/OTA 33) were retrospectively identified. Of these, 111 fractures in 106 patients (53.8% female) underwent locked plate fixation. They had an average age of 54 years (range 18 to 95 years): 34.2% were obese, 18.9% were smokers, and 18.9% were diabetic. Open fractures were present in 40.5% with 79.5% Gustilo type III. Fixation constructs for plate length, working length, and screw concentration were delineated. Nonunion and/or infection, and implant failure were used as outcome complication variables. Outcome was based on surgical method and addressed according to Pritchett for reduction, range of motion, and pain.

**Results:**

Eighty-three (74.8%) of the fractures healed after the index procedure. Twenty (18.0%) of the patients developed a NU. Four of 20 (20%) resulted in a recalcitrant NU. Length of comminution did not correlate to NU (*p* = 0.180). Closed injuries had a higher tendency to heal after the index procedure than open injuries (*p* = 0.057). Closed and minimally open (Gustilo/Anderson types I and II) fractures healed at a significantly higher rate after the index procedure compared to type III open fractures (80.0% versus 61.3%, *p* = 0.041). Eleven fractures (9.9%) developed hardware failure. Fewer nonunions were found in the submuscular group (10.7%) compared to open reduction (32.0%) (*p* = 0.023). Fractures above total knee arthroplasties had a significantly greater rate of failed hardware (*p* = 0.040) and worse clinical outcome according to Pritchett (*p* = 0.040). Loss of fixation was related to pain (*F* = 3.19, *p* = 0.046) and a tendency to worse outcome (*F* = 2.43, *p* = 0.071). No relationship was found between nonunion and working length.

**Conclusion:**

Despite modern fixation techniques, distal femoral fractures often result in persistent disability and worse clinical outcomes. Soft tissue management seems to be important. Submuscular plate insertion reduced the nonunion rate. Preexisting total knee arthroplasty increased the risk of hardware failure. Further studies determining factors that improve outcome are warranted.

## Background

Distal femoral fractures reportedly account for less than 1% of all fractures and comprise between 4%–6% of all femoral fractures [1-3]. Supracondylar femoral fractures occur commonly among two populations, young patients involved in high-energy accidents (including motor vehicle and motorcycle accidents and sports trauma) and older patients, often osteoporotic, sustaining low-energy fall fractures. Jahangir additionally described an increase of periprosthetic fractures of the distal femur in patients with previous total knee arthroplasty or distal to a total hip arthroplasty as the third common population [4].

Except in extreme circumstances, operative treatment for supracondylar femoral fractures is the standard, while nonsurgical treatment has largely fallen out of favor as the result of further advances in technique and implants [4]. Surgical fixation has consistently demonstrated better outcomes than nonsurgical management [5] mainly based on fixed angle devices starting with the blade plate, dynamic condylar screw [6,7], and nail resulting in the advent of locked plating. The current trend is toward periarticular distal femoral locking plates [8,9], which can be inserted submuscularly as a minimally invasive procedure to preserve blood supply, fracture hematoma, and avoid extensive soft tissue damage [10-13].

Definitive treatment of distal femoral fractures requires maintenance or restoration of distal femoral alignment to preserve the function of the extremity [14]. Additionally, early knee motion is central to the management of distal femoral fracture. Knee stiffness and loss of range of motion (ROM) may develop with immobilization [15], and these often contribute to a poor outcome [10]. Supracondylar fractures, intraarticular in particular, are difficult to treat to successful union without complications. Similar nonunion rates of 0%–20% for conservative treatment or internal fixation methods [5,6,16-19] have been described. This finding was treatment independent. In addition, diabetic and obese patients seem to be at high risk for healing complications, infections, and specifically nonunions [7]. Concerns have been voiced that the material of the implant might be of importance [8]. A significantly higher nonunion rate for stainless steel plate implants compared to titanium has been reported [8].

Understanding characteristics of distal femoral fractures as well as the principles and challenges of management is important in optimizing outcomes [14]. Therefore, the purpose of this study was to analyze the complications and clinical outcomes of locked plating for supracondylar femur fractures utilizing Cain's [9], Kristensen's [11], and Pritchett's [12] criterion.

## Methods

This study was an Institutional Review Board approved retrospective cohort analysis of patients undergoing LP surgical treatment for distal femoral fractures from March 2002 through June 2009 at two Level I trauma centers. The involved patients were collected from the clinical database based on a computer query of Current Procedural Terminology (CPT) codes for supracondylar fractures. All patients with supracondylar femoral fracture treated with locked plate fixation and age equal to or older than 18 years were included in this study. Patients with intramedullary fixation, metastatic disease, impaired lower extremity motor or nerve function prior to injury, and supplemental methods for bone healing were excluded.

Two hundred forty three (243) fractures were surgically treated for distal femur fractures during the study period. Sixty-one fractures were excluded because of age younger than 18 years old (14), open reduction and internal fixation other than locked plating or intramedullary fixation (40), carcinoma with metastasis to the supracondylar region (2), paraplegia (3), and implanted bone stimulator (2). Additionally, 71 were lost due to death (1), follow-up less than 6 months (62), and incomplete radiographic data (8). The death occurred during the initial hospital period and was related to other associated injuries. A final study group of 111 fractures (67 left, 44 right) in 106 patients with a mean age of 54 years (range 18–95 years) remained. There were 49 (46.2%) males and 57 (53.8%) females with an average body mass index (BMI) of 29.8 kg/m^2^ (range 17–67). Length of follow-up was 23.3 months (range 6–72). High-energy injuries were more common and occurred in 64 of 111 patients (57.7%) compared to low energy fall in 41 of 111 patients (36.9%) (Table 1). Patients with high-energy trauma averaged 44 years (range 18–88 years) while patients suffering from a low-energy fall averaged 69 years (range 31–95) (*t* = 8.27, *p* < 0.001). These injuries resulted in 72 (64.9%) closed and 39 (35.1%) open fractures. Open fractures were associated with high-energy injury mechanism (*p* < 0.001). Comorbidities and potential contributing factors were recorded (Table 2). Obesity with BMI ≥ 30 kg/m^2^ (38/111), previous total knee arthroplasty (22/111), smoker (21/111), diabetes mellitus, and a history of smoking (20/111) were most common.

**Table 1:** Mechanism of injury

**Mechanism of injury**	**Number**	**Percentage (%)**
Low energy fall	41	36.9
High energy fall	9	8.1
Motor vehicle accident	44	39.6
Motor cycle accident	7	6.3
Sport	4	3.6
Unknown	6	5.4

**Table 2:** Comorbidities and contributing factors

**Comorbidities and contributing factors (may have more than 1)**	**Number**	**Percentage (%)**
Diabetes	21	18.9
Current smoker	21	18.9
Past smoker	20	18.0
Previous total knee replacement	22	19.8
Obesity (body mass index ≥ 30 kg/m^2^)	38	34.2

**Figure 1:**
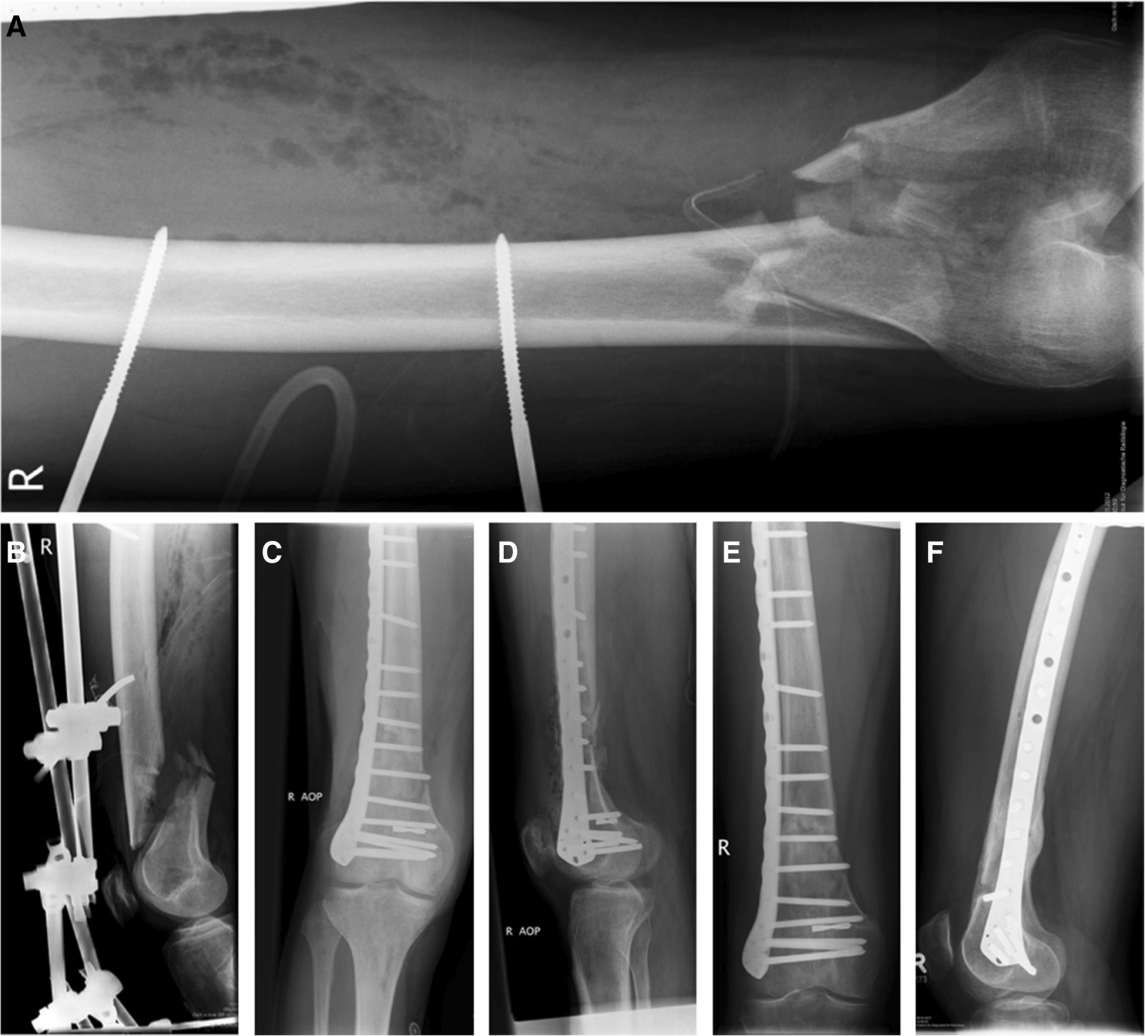
**Treatment and follow-up of a distal femoral fracture. (A)** Preoperative radiographic AP view of a distal femur fracture with external fixation. **(B)** The lateral view shows the sagittal alignment of the fragments. **(C,D)** Postoperative radiographs confirm reduction quality and implant position. **(E,F)** Callus formation and cortical continuity demonstrate ongoing fracture healing.

**Figure 2:**
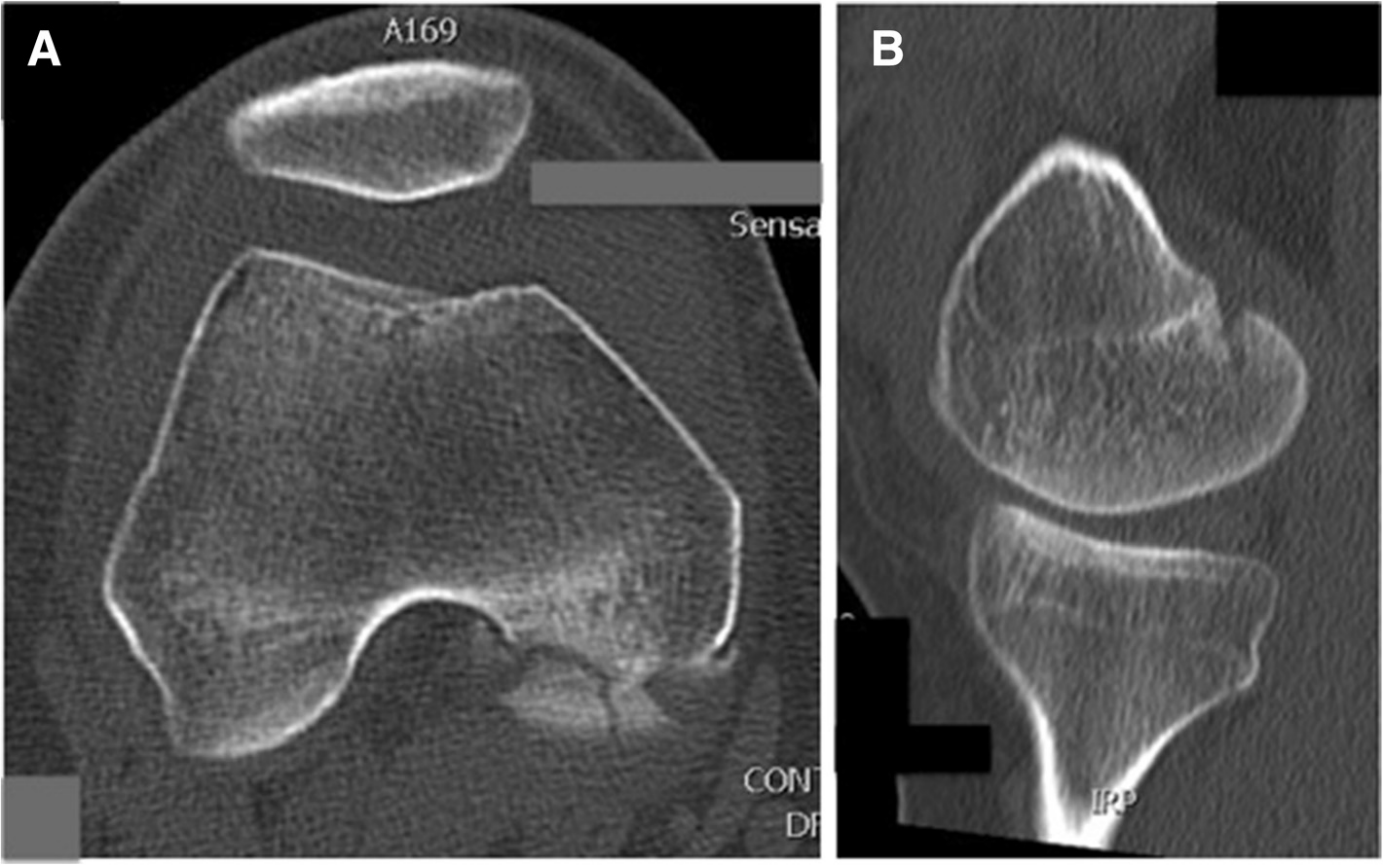
**CT-scans provide additional information concerning articular involvement. (A)** Coronal image of a Hoffa's fracture. **(B)** CT reconstruction of a Hoffa's fracture.

Each patient had two initial injury femur views (Figure 1A,B) and additional diagnostics when assumed necessary for assessing fracture pattern (Figure 2). Fractures were classified according to the AO/OTA (Arbeitsgemeinschaft Osteosynthese/Orthopaedic Trauma Association) system in 44 A-type, 4 B-type, and 63 C-type fractures [13] (Table 3). Twenty-two patients suffered from a fracture proximal to a total knee replacement (TKR). Periprosthetic fractures were additionally classified according to Lewis and Rorabeck [20].

**Table 3:** AO/OTA classification

**AO/OTA classification**	**Number**	**Percentage (%)**
33 A1	17	15.3
33 A2	5	4.5
33 A3	22	19.8
33 B1	3	2.7
33 B2	1	0.9
33 B3	0	0.0
33 C1	6	5.4
33 C2	38	34.2
33 C3	19	17.1

Open or closed reduction and internal fixation of the supracondylar femoral fracture was performed with the patient in the supine position on a radiolucent table with fluoroscopic assistance. The operative approaches to the distal femur were tailored to each patient based on the particular pattern of the injury, location of the fracture, associated injuries, and soft tissue involvement. Internal fixation of the metaphyseal part of the fracture was either performed open (36) or submuscular (75). Six trauma fellowship trained orthopedic surgeons performed the surgeries at two trauma centers. All patients had postoperative radiographs (AP, LAT) imaging to confirm reduction quality and implant position (Figure 1C,D).

Postoperatively, patients had antibiotic and deep vein thrombosis prophylaxis. Open fractures were either treated with primary closure or returned for delayed primary closure. Antibiotics were continued and readministered based upon wound severity and surgeon preference. Patients were mobilized based upon the constellation of injuries and femur fracture pattern. In general, weight bearing on the distal femoral fracture was delayed until signs of healing with callus formation or resolution of fracture lines. Formal physical therapy was instituted working on core strengthening, dynamic lumbar stabilization, range of motion, strengthening, and conditioning.

Patients were followed in the office on a regular basis at intervals of 2 weeks, 6 weeks, 12 weeks, 6 months, 1 year, and 2 years. Complaints of pain were assessed with a visual analog scale (VAS), and problems with ambulation (limp and required aides) were recorded. Clinical examination of incisional healing, motor exam, sensory exam, knee stability, range of motion (ROM), and ambulation was performed. Radiographs consisting of AP and LAT views of the distal femur were obtained and evaluated by the orthopedic surgeons during office follow-up at each interval. Additionally, all radiographs were examined digitally by two authors (MFH, SJK) utilizing a picture archiving and communication system (PACS, Kodak Carestream PACS 2006, Eastman Kodak Company, Rochester, NY, USA) and Horizon Rad Station (McKesson, Medical Imaging, San Francisco, CA, USA). Bridging of the fracture site at three cortices by callus or cortical continuity as well as obliteration of the fracture line were defined as radiographic union (Figure 1E,F) [21]. Missing radiographic evidence of fracture union with continued progress toward healing at the 6-month time point was defined as delayed union [22]. Malunion was defined as varus angulation >10° at fracture healing.

Complications were recorded concerning healing, hardware loosening, hardware failure, and revision surgery. Infection was defined as either deep or superficial. Deep infections were defined as those that required operative treatment. Superficial infections were defined as those that were treated only with local antibiotics and wound care, and no operative treatment for the infection. Complaints of leg length discrepancy, instability, and knee stiffness were recorded.

Data was analyzed using PASW® 18 (IBM, Armonk, NY, USA). Descriptive statistics including percentage, standard deviation, mean, and range were completed. Chi square and *t* tests were used to compare those that developed complications versus those that did not, based on demographic data, contributing factors, empty holes adjacent to the fracture, numbers of screws, and femoral-tibial alignment. When an unequal variance of means was present, a Wilcoxon two-sample test was used to determine differences in analysis such as proximal screw concentration in implant failure and length of comminution and nonunion. An analysis of variance (ANOVA) was used to determine a difference in categorical groups including AO/OTA classification grouped by A, B, and C, pain levels, range of motion, and outcomes categorized by the Pritchett [12] criteria. Significance was set at <0.05.

## Results

Twenty-nine fractures (26.1%) were initially stabilized using a temporary spanning external fixator. Initial temporary external fixation was commonly used with open fractures (15/39 versus 14/72, *p* = 0.029). Eight fractures of 111 (7.2%) received titanium implants while 103 fractures of 111 (92.8%) were stabilized with stainless steel implants. Seventy-five fractures of 111 (67.6%) were treated utilizing a minimal invasive submuscular approach. The implant types used are listed in Table 4.

**Table 4:** Implant types and manufacturer

**Implant type (manufacturer)**	**Frequency**	**Material**	**Percentage (%)**
Periarticular distal lateral femoral locking plate (Zimmer)	57	Stainless steel	51.4
Periloc (Smith and Nephew)	25	Stainless steel	22.5
Locked compression plate (Synthes)	21	Stainless steel	18.9
LISS (Synthes)	8	Titanium	7.2

According to the different fracture patterns, plate length varied from 6–18 holes with holes proximal to the fracture varying from 2 to 13. The number of proximal screws varied from 1 to 9. Three to five proximal screws were most common (82.9%). An average of 52% of the proximal holes were filled with screws. No difference in the proximal screw concentration for implant failure was found (*Z* = 0.4947, *p* = 0.621). Fixation of the condyles was performed with 4 to 6 screws in 90.1% of the fractures. In 33 of 111 fractures (29.7%), additional interfragmentary fixation utilizing lag screws was performed.

One hundred and one fractures (91.0%) finally healed including three malunions. Thereof, 83 fractures (74.8%) healed after the index procedure. No difference was found for healing after the index procedure when comparing titanium (7/8, 87.5%) to stainless steel (76/103, 73.8%), (*p* = 0.677). Comparing open and closed fractures, we found a greater percentage of healed fractures after the index procedure for closed injuries (80.6% versus 64.1%, *p* = 0.057). This finding becomes significant comparing closed and minimally open (Gustilo/Anderson types I and II) fractures to type III open fractures (80.0% versus 61.3%, *p* = 0.042). Fifty-two fractures (46.9%) underwent additional surgical procedures including hardware removal after fracture healing in 10 patients (9.0%) who complained of prominent medial screws. The final healing status of the patients is listed in Table 5.

**Table 5:** Healing status after distal femur fracture

**Final healing status**	**Number**	**Percentage (%)**
Healed	101	91.0
Nonunion	4	3.6
Total knee replacement	4	3.6
Antibiotic spacer after infected total knee replacement	1	0.9
Below knee amputation	1	0.9

Surgical complications were found in 14 treated fractures (12.6%). Heterotopic ossifications were removed in five patients (4.5%) and one patient developed a superficial infection, which resolved under local wound therapy and oral antibiotics. Eight patients underwent irrigation and debridement for deep infection (7.2%). Infection was related to open fracture (7/39, 18.0%, *p* = 0.003) and current smokers had a higher infection rate than non-smokers (3/21, 14.3% versus 1/51, 1.9%; *p* = 0.010), but no relationship to diabetes, implant material, or initial treatment with external fixation was found (*p* = 0.361, *p* = 0.670, and *p* = 0.203, respectively).

Of the 111 fractures, 20 (18.0%) developed a nonunion or delayed union with 11 fractures (9.9%) leading to hardware failure. Hardware failure occurred proximally in six cases, three plates fractured in the area of the working length and two plates loosened distally. Postoperative staged bone grafting was performed in 19 patients. Four fractures underwent planned staged bone grafting with one fracture requiring an additional second bone grafting. A significant reduction of nonunion formation was found in the submuscular minimal invasive group (10.7%) compared to the open reduction group (32.0%) (*p* = 0.024). Length of comminution did not influence nonunion rate (*Z* = 1.3406, *p* = 0.180). No difference in working length was found in fractures resulting in nonunions compared to fractures with primary healing (*p* = 0.784). Additional lag screws did not influence nonunion rate (*p* = 0.590).

Hardware failure was related to nonunion (*p* < 0.001). Fractures proximal to total knee arthroplasties had a significantly greater rate of failed hardware (*p* = 0.040). No difference in hardware failure was found comparing titanium and stainless steel (*p* = 0.948). Additional lag screws did not influence hardware failure (*p* = 0.731).

Alignment was restored to an average of 7.4° of valgus (range −4.4° to 16.3°) and 87.8° of extension (range 71°–118°). The loss of fixation was an average 0.97° (range 0° to 14°). No significant difference was found in loss of fixation for patients with lag screws compared to patients without lag screws (*Z* = 0.1039, *p* = 0.917).

At the last follow-up, 47.8% of the patients did not complain of any pain (VAS 0). Thirty-seven percent had mild (VAS 1–3), 10.8% had moderate (VAS 4–6), and 1.8% (2 patients) had severe (VAS 7–10) pain. No relationship between open fractures and persistent pain was found (*p* = 0.178). Pain was not related to healing status (*p* = 0.698), valgus alignment (*p* = 0.759), or range of motion (*p* = 0.214). Patients with increased loss of fixation had higher pain levels (*F* = 3.19, *p* = 0.046).

Patients had reduced range of motion resulting mostly from loss of flexion. Extension was restored to a mean loss of 1.4°. Seventeen knees (15.3%) had an extension deficit of 5° or more. Flexion ranged from 0° in the patient with explanted TKR to 150° with a mean flexion of 114°. One hundred three knees (92.8%) were able to flex to 90° according to Cain's criteria [9]. Additionally, flexion has been divided into four groups (<60, 60–94, 95–104, >104) according to Kristensen [11] (Table 6). Utilizing Kristensen's criteria, 75.7% of the patients had acceptable flexion. This was not influenced by AO/OTA classification (*F* = 1.05, *p* = 0.354) or AP alignment (*t* = 0.12, *p* = 0.905). Reduced flexion was found in patients with advanced age and periprosthetic fractures (*t* = −3.32, *p* = 0.001, *Z* = −2.366, *p* = 0.018, respectively).

**Table 6:** **Clinical outcome (range of motion) according to Kristensen**[11]

	**Range of motion**				
	**<60°**	**60–94°**	**95–104°**	**>104°**	**Unknown or not applicable**
Number of patients	3	19	11	73	5
Percentage (%)	2.7	17.1	9.9	65.8	4.5

Combining the results of pain, deformity, and range of motion for outcome using the rating system of Pritchett [12] (Table 7), we had 22 excellent (20.8%), 29 good (27.4%), 48 fair (45.3%), and 7 poor (6.6%) results. Five patients were not classifiable due to TKR and antibiotic spacer. Age or mechanism of injury did not influence the outcome (*F* = 1.03, *p* = 0.382; *p* = 0.341, respectively), but patients with poor outcome had a significantly higher BMI than patients with excellent outcome (*F* = 4.17, *p* = 0.008). Comparing AO/OTA classification to outcome did not reveal any difference (*p* = 0.420). A significantly worse outcome was found for patients with periprosthetic fractures (*p* = 0.040). Patients with varus malalignment did not have a different outcome (*F* = 1.39, *p* = 0.250), but greater loss of fixation seems to trend toward a worse outcome (*F* = 2.43, *p* = 0.071). No difference was found comparing the outcome of submuscular procedures to open reduction (*p* = 0.899).

**Table 7:** The Pritchett rating system for supracondylar femoral fractures

**Result**	**Criteria**
Excellent	Full extension; flexion >110°; no deformity or joint incongruity
Good	Full extension; flexion >90°; <5° of varus or valgus; loss of length <1.5 cm, minimal pain
Fair	Flexion of 75°–90°; varus, valgus, or angular deformity of 5°–10°; mild or moderate pain
Poor	Flexion <75°; valgus, varus, or angular deformity >10°; articulate incongruity; frequent pain requiring analgesics

## Discussion

Controversy still exists regarding the surgical treatment method of distal femoral fractures. Internal fixation procedures are dependent on fracture type and the surgeon's preference. While intramedullary nails have comparable advantages as locking plates such as percutaneous placement, indirect fracture reduction, soft tissue protection, success in osteoporotic bone, and high healing rates [23], locking plates have become the most commonly used method to stabilize fractures of the distal femur [24]. Advanced age of the patient population might be a reason. Improved distal fixation for locked plates compared to blade plate and retrograde nailing has been demonstrated in osteoporotic bone [25]. Although locking plates have provided a valuable additional option for treatment of distal femoral fractures, the use of locked plates has expanded and the numbers of fractures fixed with these plates have increased, complications related to slow healing including nonunion, delayed union, and implant failure are not infrequent and are ongoing problems in managing these fractures [17,24].

Earlier studies have shown reduced nonunion rates for locked plating of distal femoral fractures compared to non-locking plates [5,26], but more recent studies found nonunion rates up to 20% [17-19]. In the current study, 18% of the fractures showed signs of delayed or non-union. Multiple reasons influence union rates. Higher stiffness of locking plates has been related to suppressing interfragmentary movement and callus formation [17,27]. But in a systematic review by Zlowodski [28] comparing traditional plating, intramedullary nails, and locking plates, no observed differences were found between implants regarding the rate of nonunion, infection, fixation failure, or revision surgery [23]. Titanium has been noted to have superior biocompatibility with an elasticity modulus more similar to bone than stainless steel [29]. Therefore, increased stiffness of stainless steel implants was related to higher nonunion rates [8]. Yet, this was based on unpublished data. Additionally, no significant difference for closed fractures was found. The significance was based on open fractures. On the contrary, biomechanical testing demonstrated only a significantly greater stiffness for torsion in stainless steel plates (LISS) [29]. A different study by Henderson found no significant difference between non-union rates for stainless steel or titanium (*p* = 0.71) [18]. The current study did not discover any difference for nonunion rates or hardware failure between titanium and stainless steel. Conclusions are not definitive due to insufficient sample size. A power analysis considering significance of 0.05 and power of 0.80 requires a sample of 642 fractures equally distributed between hardware metal based on Henderson's data.

Axial stiffness and torsional rigidity of internal fixation is mainly influenced by working length [30]. There is a fine line between flexible fixation, which enhances callus formation and improves the healing process, and an unstable fixation, which leads to nonunion and/or implant failure [30]. Short-spanning segments concentrate the stress moment and may lead to failure of the construct [31]. Henderson found no empty holes next to the fracture in 71% of the nonunions [18]. Bottlang reported a 19% nonunion rate in a cohort of 72 patients but found no significant difference in bridging span in those that healed compared with those that did not heal [32]. The current recommendation for adequate bridge plate fixation is three or four empty holes at the level of the fracture [33]. We found a shorter working length in patients with nonunions. Additional lag screws did not influence the nonunion rate and did not reduce loss of fixation.

The recommended screw ratio is 0.4 to 0.5 for bridging fixation with three to four screws on either side of the fracture gap [24,30,34]. Ricci recommended at least five screws proximally but required an adequate plate length to maintain screw density below 60% [35]. In our study, these recommendations were followed. More than 82% had three to five proximal screws and only 52% of the proximal holes were filled. This may be the reason why we did not see differences in these parameters for nonunion or hardware failure.

Distal femoral alignment is one of the treatment priorities. The femoral shaft is oriented 7° of valgus in relation to the knee joint [36]. Maintaining this alignment is critical to the function and durability of the limb [14]. Coronal plane alignment has been shown to be the most difficult factor to control and the most crucial to overall outcome [37]. Malalignment in the axial and sagittal planes also affects knee kinematics and range of motion [14]. When comminution is present, supracondylar femoral fractures are especially prone to varus collapse [38]. The current study supports the reduced rate of fixation loss due to the utilization of locked plating and shows that additional lag screws do not influence varus collapse. Patients with greater loss of fixation tend to have a worse outcome.

We found more than 40% open fractures in our study population. Previous studies stated that open fractures are common in the setting of distal femur fractures (19%–54%) [17]. Open fractures were related to high-energy injury mechanism and a greater prevalence of infection. Therefore, the outcome of distal femoral fractures, similar to other major injuries, not only depends on bony reconstruction but also on soft tissue management. Henderson states, ‘The diversity of injury patterns and bone quality and the complex mechanical and biological interplay in each individual case make it difficult to separately assess and study potentially important variables’ [17]. The importance of soft tissue preservation for fracture healing has been previously described. We confirmed that submuscular plate insertion reduces nonunion formation significantly.

Outcome has been previously defined by reduction quality, range of motion, and pain [9,11,12]. Historically, different classification systems have been utilized. Following these, we found 92.8% good flexion according to Cain [9] and 75.7% of the patients had acceptable flexion following the criteria of Kristensen [11]. Utilizing the more strict criteria of Pritchett [12], only 45.9% excellent or good results were achieved. Multiple factors are related to patient outcome. We showed that outcome was associated with obesity and periprosthetic fractures. From a surgical and a mechanical standpoint, submuscular procedures influence nonunion rate but not the final outcome. Additionally, patients with varus malalignment did not have a different outcome, but greater loss of fixation was related to worse outcome.

The limitations of the study are related to the retrospective design. Almost 16.5% of the initial patient cohort was excluded due to operative fixation techniques other than locked plating which may have created a selection bias. Furthermore, the majority of fractures in this study were treated utilizing stainless steel plates. No comparison between the Pritchett functional outcome and subjective outcome scores were performed. The strength of this study is the large number of patients from two Level 1 trauma centers treated similarly by fellowship-trained orthopedic trauma surgeons. In addition, the majority of fractures were treated with similar plate lengths, screw concentration, and working lengths.

## Conclusion

Despite modern fixation techniques of locked periarticular plating, distal femoral fractures often still result in persistent disability and poor clinical outcome. Soft tissue management seems to be important. Submuscular plate insertion reduces the nonunion rate. Preexisting total knee arthroplasty increases the risk of hardware failure. Further studies determining factors that improve outcome are warranted.

## Competing interests

The authors declare that they have no competing interests.

## Authors’ contributions

MFH performed the data collection for the first study site, participated in its design, carried out the literature search, performed the data interpretation, and drafted the manuscript. DLS performed the statistical analysis and was involved in revising the manuscript. SJK carried out the data collection for the second study site. CBJ and PT3 initiated the study, were involved in revising it critically for important intellectual content, and have given final approval of the version to be published. All authors read and approved the final manuscript.
